# Differences in Race Characteristics between World-Class Individual-Medley and Stroke-Specialist Swimmers

**DOI:** 10.3390/ijerph192013578

**Published:** 2022-10-20

**Authors:** Tomohiro Gonjo, Marek Polach, Bjørn Harald Olstad, Michael Romann, Dennis-Peter Born

**Affiliations:** 1 Department of Rehabilitation & Sport Sciences, Bournemouth University, Poole BH12 5BB, UK; 2 Faculty of Physical Culture, Palacký University Olomouc, 771 47 Olomouc, Czech Republic; 3 Department of Competitive Swimming, Czech Swimming Federation, 160 17 Prague, Czech Republic; 4 Department of Physical Performance, Norwegian School of Sport Sciences, 0863 Oslo, Norway; 5 Department for Elite Sport, Swiss Federal Institute of Sport Magglingen SFISM, 2532 Magglingen, Switzerland; 6 Section for High-Performance Sports, Swiss Swimming Federation, 3063 Bern, Switzerland

**Keywords:** swimming, race analysis, performance analysis, elite swimmers

## Abstract

The purpose of the present study was to investigate differences between world-class individual medley (IM) swimmers and stroke-specialists using race analyses. A total of eighty 200 m races (8 finalists × 2 sexes × 5 events) at the 2021 European long-course swimming championships were analysed. Eight digital video cameras recorded the races, and the video footage was manually analysed to obtain underwater distance, underwater time, and underwater speed, as well as clean-swimming speed, stroke rate, and distance per stroke. Each lap of the IM races was compared with the first, second, third, and fourth laps of butterfly, backstroke, breaststroke, and freestyle races, respectively. Differences between IM swimmers and specialists in each analysed variable were assessed using an independent-sample *t*-test, and the effects of sex and stroke on the differences were analysed using a two-way analysis of variance with relative values (IM swimmers’ score relative to the mean specialists’ score) as dependent variables. Breaststroke specialists showed faster clean-swimming speed and longer distance per stroke than IM swimmers for both males (clean-swimming speed: *p* = 0.011; distance per stroke: *p* = 0.023) and females (clean-swimming speed: *p* = 0.003; distance per stroke: *p* = 0.036). For backstroke and front crawl, specialists exhibited faster underwater speeds than IM swimmers (all *p* < 0.001). Females showed faster relative speeds during butterfly clean-swimming segments (*p* < 0.001) and breaststroke underwater segments than males (*p* = 0.028). IM swimmers should focus especially on breaststroke training, particularly aiming to improve their distance per stroke.

## 1. Introduction

In competitive swimming, swimmers compete in four different strokes, namely butterfly, backstroke, breaststroke, and freestyle (front crawl). Individual medley (IM) is a unique event in which swimmers perform all four strokes in the abovementioned order, meaning that they are required to have advanced underwater and above-water swimming skills in all four strokes. In butterfly, backstroke, and freestyle races, swimmers are allowed to perform underwater locomotion up to the 15 m mark [1,2], and elite swimmers usually break the water surface at around 10–15 m after the start and 5–12 m following turns [1,2]. Although there is no limit for underwater locomotion distance in breaststroke, swimmers are only permitted to perform one dolphin kick and one arm and leg cycle during the underwater phase [3], resulting in underwater distances of 11–15 m after the start and 8–10 m after turns for elite swimmers [1,2]. The same rules are applied to IM events; thus, it is reasonable to assume that IM swimmers should ideally have similar underwater and above-water performance as stroke-specialists. However, it is currently unclear how similar or different IM and specialist swimmers’ performances are.

The lack of evidence regarding differences between IM swimmers and stroke-specialists is a result of the absence of IM race analysis. Race analysis is a method for objectively quantifying swimmers’ performance by dividing a race into smaller segments and quantifying kinematic parameters for each phase. It allows researchers and practitioners to evaluate the performance as a whole in real race conditions rather than assessing various skills (i.e., start, turn, clean swimming, and underwater swimming) separately in an experimental lab environment. In the last decade, many researchers conducted race analyses of international swimming races, such as the European [1,2] and World Championships [4,5,6], to assess world-class swimming performance in the four strokes of both sexes. However, detailed information on IM races are limited [7]. 

To identify the performance difference between different groups of swimmers, ideally, the performance of the same events should be compared, e.g., comparing 100 m stroke performance between IM swimmers and specialists. However, such comparisons between IM swimmers and stroke-specialists are challenging due to the limited number of IM swimmers who join single-stroke events. An alternative option would be to compare 200 m IM races and specific laps in 200 m single stroke events. Several studies have focused on world-class IM races using public information, such as the race time and lap times. However, these studies only analysed the pacing strategy of IM swimmers [8], the relationships of race times in IM and single stroke events [9], or the difference between short course and long course IM races [10]; however, no studies have compared race analyses between IM swimmers and stroke-specialists. 

When investigating the differences between IM swimmers and specialists, it is necessary to consider the effect of sex, because female swimmers spend a larger percentage (0.3%) of the 200 m IM race on swimming breaststroke and a smaller percentage on front crawl (0.3%) compared to male swimmers [8]. Despite the small difference in this percentage, given that 200 m IM in elite male and female swimmers lasts around 115–130 s, the 0.3% difference corresponds to 0.3–0.4 s, which is considered meaningful in elite swimming performance, as even one-hundredth of a second can differentiate swimmers’ rankings in competitions. Therefore, the results of Saavedra et al. [8] imply that the strategies of male and female IM swimmers are different, and trends may differ depending on sex when comparing IM swimmers and stroke-specialists. 

Given the lack of evidence on the differences between IM swimmers and stroke-specialists, comparing the race performance between the IM and specialist groups would be beneficial. Identifying similarities and differences between IM swimmers and specialists would be valuable, particularly for IM swimmers, as the evidence would inform coaches and swimmers about which elements IM swimmers should focus on during their training. Therefore, the purpose of the present study was to investigate differences between world-class IM and stroke-specialists by comparing each lap of 200 m IM and the corresponding lap in 200 m stroke events. The null hypotheses of the present study were that there would be no effects of sex and stroke on the difference between IM and stroke events. 

## 2. Materials and Methods

### 2.1. Participants

A total of eighty races, including forty male (age: 24.2 ± 3.6 and FINA points: 906 ± 37) and forty female (age: 23.9 ± 4.4 and FINA points: 880 ± 39) 200 m finalists at the 2021 European long-course swimming championships in Budapest were analysed. From each final (IM, butterfly, backstroke, breaststroke, and freestyle for both males and females) data of all eight swimmers were used to compare race characteristics between IM swimmers and stroke-specialists. Participants of the European swimming championships are video monitored for television broadcasting and race analyses by the Ligue Européenne de Natation (LEN), which is the organiser of the event. The present study was conducted as a part of a larger investigation project [11], which was preapproved by the institutional review boards of the Swiss Federal Institute of Sport Magglingen (Reg.-Nr. 140_LSP_072021) and conducted according to the ethical principles for medical research involving human subjects of the World Medical Association (WMA Declaration of Helsinki).

### 2.2. Data Collection

The finals were recorded with eight cameras at 50 Hz sampling frequency (2× XAVC S, Sony Group Corporation, Minato, Japan, 5× HC-X1000 and 1× HC-X1500, Panasonic Corporation, Kadoma, Japan). The cameras were mounted about 20 m from and 5 m above the pool deck. The lane ropes were marked every 10 m between the 5 and 45 m point, and a virtual line for each lane was established by connecting the corresponding points on the two ropes of the lane at 15 m, 35 m, and 45 m. Furthermore, as the lane ropes consisted of 0.1 m floats, the same virtual line approach was applied to every 0.1 m between 5 m to 15 m to quantify the head breakout distance and time for each swimmer. Video footage was manually analysed with Kinovea 0.9.1 (Joan Charmant & Contrib., kinovea.org). Time stamps were exported to a specific spreadsheet (Excel 365, Microsoft Corporation, Redmond, DC, USA) to calculate split times and stroke parameters. Inter-rater reliability for breakout time, breakout distance, swimming speed, stroke rate, and distance per stroke showed a mean intra-class correlation coefficient of 0.99 [11].

### 2.3. Data Processing and Analysis

For the comparison between IM swimmers and stroke-specialists, data from the first, second, third, and fourth laps were selected from the 200 m butterfly, backstroke, breaststroke, and freestyle events, respectively. Clean-swimming speed was determined as distance divided by duration from the breakout point to the 45 m mark of that particular lap. Stroke rate (cycles/min) was measured twice per lap, at the 15 m and 35 m marks, and the average was used for statistical analysis. Distance per stroke (m/cycle) was computed as the clean-swimming speed divided by the stroke rate and multiplied by 60. The underwater time was defined as the duration from the end of the previous lap until the head breakout, and the underwater speed was calculated as the underwater distance divided by underwater time. The obtained variables in each IM swimmer were expressed as both absolute and relative values. The relative value for each variable was quantified as:

(1) $$rVAR_{rel}=\frac{VAR_{abs}-VAR_{s\_mean}}{VAR_{s\_mean}}\;\cdot 100,$$

where *VAR_rel_* and *VAR_abs_* are the relative and absolute value for IM swimmers, respectively, and *VAR_s_mean_* represents the between-participant mean of the specialist group in the same variable.

### 2.4. Statistical Analysis

The Shapiro–Wilk test was used to check the normality of all datasets. The absolute values of the obtained variables for IM swimmers and stroke-specialists were compared with an independent sample *t*-test. Non-normally distributed data were compared using the Mann–Whitney U test. The *p*-values were corrected using the Holm–Šídák method to account for multiple comparisons of each variable. The central tendency of the data was expressed as the mean and standard deviation (SD) for normally distributed data and the median and interquartile range for data with a non-normal distribution. For each pair, Cohen’s d or r-value was calculated for parametric and non-parametric comparisons, respectively. The thresholds for small, medium, and large effects were set at 0.2, 0.5, and 0.8 for Cohen’s d, and 0.1, 0.3, and 0.5 for r-values [12].

The effect of sex and stroke on the difference between IM swimmers and stroke-specialists, together with the partial eta square (*η_p_*^2^), were analysed by a two-way analysis of variance (ANOVA) for each obtained variable using the relative value as the dependent variable. The Box–Cox transformation was applied to the dataset if the analysis included any non-normally distributed data [13]. When a significant effect of strokes or a significant interaction between stroke and sex was observed, post hoc comparisons were performed with the Holm–Šídák correction. All statistical tests were performed using GraphPad Prism 9.3.1 (GraphPad Software, San Diego, CA, USA) with alpha = 0.05. 

## 3. Results

Descriptive statistics for the absolute variables, as well as the results from the *t*-tests, are displayed in Table 1. In both male and female swimmers, breaststroke specialists showed a 3–4.5% faster clean-swimming speed (*p* ≤ 0.011; *d* = 2.53 for males and *r* = 0.70 for females) than IM swimmers. In male swimmers, the clean-swimming speed of front crawl specialists was about 2% faster compared to IM swimmers (*p* = 0.012; *d* = 2.41), and female IM swimmers showed a 4% higher butterfly clean-swimming speed than butterfly specialists (*p* < 0.001; *r* = 0.84). In both male and female breaststroke-specialists, distance per stroke was about 17% longer than that of IM swimmers (*p* ≤ 0.036; *d* ≥ 2.14), while the breaststroke stroke rate did not differ between the groups. No differences were found between stroke-specialists and IM swimmers in stroke rate and distance per stroke for the other strokes. Both male and female stroke-specialists showed 14–37% faster underwater speed in backstroke and front crawl than IM swimmers (*p* < 0.001; *r* = 0.84 for male backstroke and *d* ≥ 3.64 for the others). However, when focusing on the breakout distance and time in these two strokes, no significant differences were found, except for a 26% shorter underwater time in male front crawl specialists than in male IM swimmers (*p* = 0.013; *d* = 2.40). Male butterfly specialists showed a 6% longer underwater time than male IM swimmers (*p* = 0.017; *r* = 0.68), and female breaststroke-specialists exhibited an underwater speed about 5% slower compared to female IM swimmers (*p* = 0.029; *d* = 1.97).

The two-way ANOVA showed significant interactions between sex and stroke for clean-swimming speed and underwater speed (Table 2). Significant stroke effects were observed for all other variables except for underwater distance (Table 2). No significant sex effects were identified in any of the analysed variables. 

**Figure 1:**
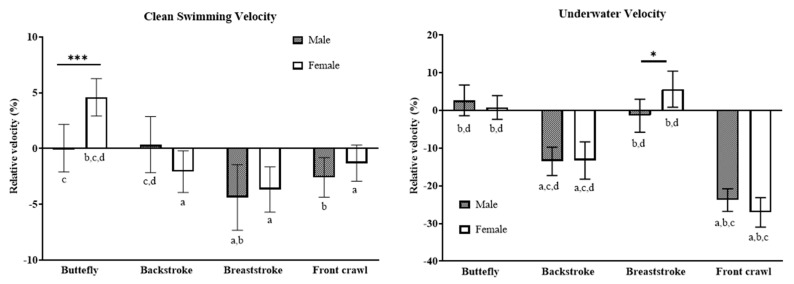
Results from between-sex comparisons as well as between-stroke comparisons (post hoc test) for the clean-swimming velocity and underwater velocity. * and ***, respectively, show a significant sex difference corresponding to *p* < 0.05 and *p* < 0.001. a, b, c, and d show a significant difference (*p* < 0.05) to butterfly, backstroke, breaststroke, and front crawl, respectively.

**Figure 2:**
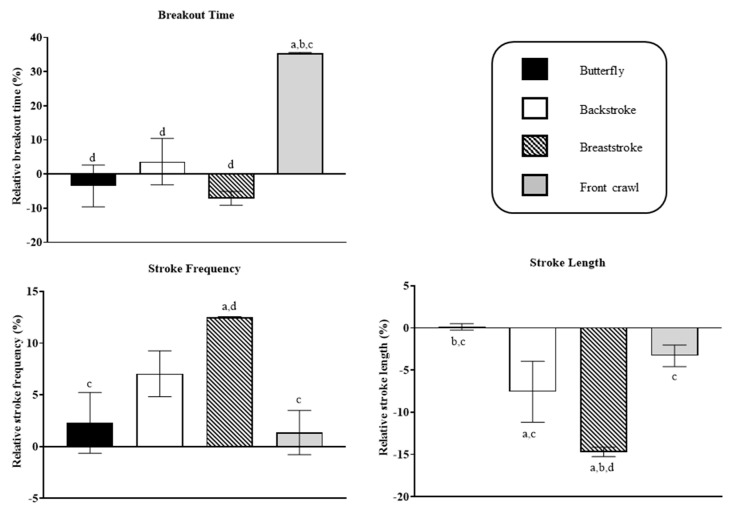
Results from between-stroke comparisons (post hoc test) for stroke rate, distance per stroke, and breakout time. a, b, c, and d show a significant difference (*p* < 0.05) to butterfly, backstroke, breaststroke, and front crawl, respectively.

Results from the post hoc test of the two variables with a significant interaction are displayed in Figure 1. The relative butterfly clean-swimming speed in female IM swimmers was 5% higher than that in male IM swimmers (*p* < 0.001; *d* = 2.38), as was the relative underwater speed during breaststroke (*p* = 0.028; *d* = 1.53). As for the between-stroke comparison in clean-swimming speed, relative butterfly speed was significantly higher than relative speed of the other three strokes in females (*p* < 0.001; *d* ≥ 3.57). In male swimmers, the relative breaststroke speed was slower compared to butterfly and backstroke (*p* < 0.001; *d* = 1.72 and 1.73, respectively), and the relative speed in front crawl was lower than in backstroke (*p* = 0.036; *d* = 1.35). Post hoc test results of the three variables that only showed a significant stroke effect are also shown in Figure 2. The relative breakout time was longer for front crawl than for the other three strokes (*p* < 0.001; *d* ≥ 1.58). The relative stroke rate in breaststroke was higher than in butterfly (*p* = 0.002; *d* = 1.11) and front crawl (*p* < 0.001; *d* = 1.27), and breaststroke also showed longer relative distance per stroke than butterfly (*p* < 0.001; *d* = 2.09), backstroke (*p* = 0.013; *d* = 0.99), and front crawl (*p* < 0.001; *d* = 1.68). Furthermore, the relative distance per stroke in backstroke was shorter compared to butterfly (*p* < 0.001; *d* = 1.33). 

## 4. Discussion

The present study aimed to investigate differences between IM swimmers and stroke-specialists. Both male and female breaststroke-specialists showed a 3.0–4.5% faster clean-swimming speed during the third lap of their 200 m race compared to IM swimmers’ breaststroke lap. Swimming speed is the product of distance per stroke and stroke rate [14,15], and swimmers generally control their speed by increasing stroke rate rather than distance per stroke [16,17]. In other words, higher stroke rate increases exercise intensity, thus energy cost, which is the energy expenditure required to swim a given distance [16]. However, there were no significant differences in stroke rate between breaststroke specialists and IM swimmers, and both male and female breaststroke specialists showed longer distance per stroke. Thus, it is reasonable to assume that breaststroke-specialists are technically more efficient and can produce greater propulsion than IM swimmers. From the relative stroke rate and distance per stroke results, it is evident that differences between IM swimmers and stroke-specialists in breaststroke are greater than in the other strokes, implying that the primary difference between IM swimmers and specialists is breaststroke. This result implies that IM swimmers can benefit from improving breaststroke performance by increasing their distance per stroke. There are several factors which affect the distance per stroke in breaststroke. For example, a previous study reported that elite swimmers showed faster swimming speeds during arm stroke compared with non-elite swimmers, despite their intra-cycle minimum velocity being similar [18]. Another study [19] exhibited a significant correlation between 50 m breaststroke velocity and propulsive impulse produced by the feet (assessed by pressure sensors), which implies the importance of the feet producing a large propulsive force over a period of time. These two studies demonstrate the importance of propulsive limb motions in breaststroke, and therefore it might be beneficial for IM swimmers to focus on improving propulsive techniques in breaststroke (rather than focusing on reducing the resistive force) to achieve a longer distance per stroke. For this purpose, strength training might also be beneficial, as it has been reported that swimmers can benefit from low volume, high velocity/force strength training to improve their distance per stroke [20]. However, it should be noted that this strength training recommendation is based on reviewing and summarising studies that focused on front crawl swimming. Therefore, future studies should investigate the effect of strength training on distance per stroke in breaststroke swimming. 

Even though male front crawl specialists and female butterfly specialists showed faster and slower clean-swimming speed than IM swimmers, respectively, no differences were observed in stroke rate and distance per stroke. These results indicate that the differences in clean-swimming speed were probably associated with non-technical factors, such as swimmers’ pacing strategies. As relative clean-swimming velocities in the last two IM strokes were slower than the first two strokes in both sexes, IM swimmers might have selected a more pronounced positive pacing strategy compared to stroke-specialists. A previous study [8] that investigated relative lap time (lap time normalised by the total race time) in IM races suggested that male swimmers tended to utilise a more pronounced positive race pace strategy than female swimmers. However, the previous study only focused on lap times, and the physiological differences between males and females were not considered. In general, females are more fatigue-resistant than males [21,22], meaning that even if male and female swimmers invest the same amount of effort at the beginning of the race, it is likely that males would be more fatigued than females and exhibit slower relative time at the end of the race. Therefore, the relative lap time difference between males and females reported in the previous study might be a consequence of an inter-sex physiological difference rather than distinct pacing strategies. In the current study, the difference between IM swimmers and butterfly specialists in clean-swimming speeds was greater in females than males; there was no difference between male IM swimmers and specialists (0.00 m/s, *d* = 0.04), while female IM swimmers showed a greater clean-swimming speed of 0.07 m/s (*r* = 0.84). Therefore, contrary to the suggestion in the previous study [8], female IM swimmers might in fact adopt a more pronounced positive pacing strategy, i.e., spending more energy at the beginning of the race with a greater decline in velocity throughout the race, than male IM swimmers. Nevertheless, the present study did not take any physiological measures into account either. This is a limitation of the study, as it is probable that swimmers have different physiological responses associated with distinct exercise patterns (maintaining a single stroke throughout the race or combining different strokes). Therefore, further studies should apply a lap-to-lap physiological assessment, as was undertaken in a recent laboratory setting for 200 m front crawl [23], to investigate potential differences in the pacing strategies between stroke-specialists and IM swimmers, as well as between male and female IM swimmers. Such studies should be conducted in experimental conditions rather than in actual races because, during competitions, research is always limited to methods that do not interfere with the swimmers’ performance.

Stroke-specialists showed faster underwater speeds in front crawl and backstroke than IM swimmers. Consequently, the relative underwater velocities in these two strokes were slower than those of butterfly and breaststroke in IM swimmers, which may be due to differences in turning techniques. In IM races, there is a time gap between the start of the lap and foot contact with the wall in front crawl and backstroke. However, in specialists’ backstroke and front crawl races, the lap is initiated the moment the feet touch the wall. It is currently unclear how much time swimmers spend between the hand touch and foot touch (pivot time) during the butterfly–backstroke and breaststroke–front crawl turns. However, as swimmers utilise the open-turn technique in both IM turns, it is reasonable to assume that the pivot times in these IM turns are similar to those in butterfly and breaststroke races. Previous studies have reported that pivot times in butterfly and breaststroke races are about 0.86–1.03 s [24,25]. When comparing the underwater speed results between the specialists and IM swimmers in front crawl and backstroke, the difference is 13.3%, 27.1%, 13.5%, and 12.8% in female backstroke, female front crawl, male backstroke, and male front crawl, respectively. However, assuming pivot times of 0.8 s in IM turns, these differences become −6.8%, −3.3%, −1.3% and 1.6%, which supports the possibility of pivot times affecting the results. 

A similar argument may explain the significantly faster underwater speed and slightly shorter mean underwater time (albeit non-significant) in female IM swimmers compared to breaststroke-specialists. Chainok et al. [26] investigated different types of backstroke–breaststroke turns and reported that the new crossover turn technique, commonly used by IM swimmers in recent years, significantly improved push-off speed compared with other turning techniques. However, Choinok et al. only tested age-group swimmers, and our study did not detect any difference in the breaststroke underwater speed in males; therefore, further studies are needed to investigate differences in turn parameters and underwater locomotion between IM swimmers and breaststroke-specialists. 

## 5. Conclusions

In conclusion, IM swimmers show similar performance in butterfly, backstroke, and front crawl to stroke-specialists in 200 m races. However, breaststroke-specialists’ performance in 200 m races (lap 3) is better than that of IM swimmers, due to a longer distance per stroke. Assuming that elite stroke-specialists’ outcomes are benchmarks of elite swimming performance, this result implies that IM swimmers have the potential to improve more in breaststroke than in the other three strokes. In other words, focusing on breaststroke training would benefit IM swimmers’ performance. Both males and females generally showed a similar trend in the difference between IM swimmers and specialists, except for faster clean-swimming speed in lap 1 (butterfly) in female IM swimmers compared to butterfly specialists. This difference was not observed in male swimmers.

## Figures and Tables

**Table 1:** Descriptive statistics and results from an unpaired *t*-test. Values in italic font show the median (interquartile range) for non-normally distributed data.

		Males						Females					
		Medley Swimmers		Stroke-Specialists		*p*-Value	Effect Size	Medley Swimmers		Stroke-Specialists		*p*-Value	Effect Size
		Mean	(SD)	Mean	(SD)			Mean	(SD)	Mean	(SD)		
Butterfly	Clean-swimming speed (m/s)	1.81	(0.04)	1.81	(0.03)	0.978	*d* = 0.04	1.65	(0.03)	*1.58*	*(0.02)*	<0.001	*r* = 0.84
	Stroke rate (cycles/min)	*51.77*	*(2.99)*	50.85	(1.91)	0.968	*r* = 0.07	53.96	(3.67)	51.71	(2.49)	0.314	*d* = 1.01
	Distance per stroke (m/cycle)	2.25	(0.11)	2.25	(0.09)	0.996	*d* = 0.00	1.84	(0.12)	1.83	(0.10)	0.898	*d* = 0.10
	Underwater distance (m)	12.78	(0.54)	13.51	(0.34)	0.024	*d* = 2.28	12.38	(1.12)	12.05	(1.09)	0.811	*d* = 0.42
	Underwater time (s)	*4.87*	*(0.36)*	5.18	(0.21)	0.017	*r* = 0.68	5.23	(0.51)	*5.41*	*(0.56)*	0.980	*r* = 0.01
	Underwater speed (m/s)	2.68	(0.11)	2.61	(0.06)	0.253	*d* = 1.13	2.37	(0.07)	*2.27*	*(0.10)*	0.130	*r* = 0.39
Backstroke	Clean-swimming speed (m/s)	1.63	(0.04)	1.62	(0.04)	0.949	*d* = 0.18	1.45	(0.03)	1.49	(0.04)	0.168	*d* = 1.30
	Stroke rate (cycles/min)	41.56	(1.35)	39.40	(2.55)	0.196	*d* = 1.50	42.43	(3.13)	39.06	(3.84)	0.209	*d* = 1.36
	Distance per stroke (m/cycle)	2.41	(0.09)	2.54	(0.14)	0.155	*d* = 1.48	2.07	(0.15)	2.30	(0.21)	0.070	*d* = 1.79
	Underwater distance (m)	11.14	(1.75)	11.92	(1.92)	0.653	*d* = 0.60	7.91	(1.39)	9.18	(1.46)	0.334	*d* = 1.26
	Underwater time (s)	*6.12*	*(1.47)*	5.31	(0.87)	0.549	*r* = 0.24	4.39	(0.76)	4.44	(0.88)	0.989	*d* = 0.09
	Underwater speed (m/s)	1.94	(0.08)	*2.22*	*(0.12)*	<0.001	*r* = 0.84	1.80	(0.10)	2.08	(0.11)	<0.001	*d* = 3.64
Breaststroke	Clean-swimming speed (m/s)	1.41	(0.04)	1.47	(0.03)	0.011	*d* = 2.53	1.28	(0.03)	*1.32*	*(0.03)*	0.003	*r* = 0.70
	Stroke rate (cycles/min)	*37.04*	*(6.42)*	34.62	(3.76)	0.221	*r* = 0.47	38.98	(3.72)	34.67	(3.52)	0.122	*d* = 1.68
	Distance per stroke (m/cycle)	2.21	(0.21)	2.60	(0.27)	0.023	*d* = 2.30	1.98	(0.21)	2.32	(0.23)	0.036	*d* = 2.14
	Underwater distance (m)	10.18	(0.81)	10.96	(0.92)	0.257	*d* = 1.27	8.30	(0.94)	8.62	(0.55)	0.800	*d* = 0.60
	Underwater time (s)	5.93	(0.49)	6.28	(0.46)	0.286	*d* = 1.06	5.11	(0.75)	5.59	(0.40)	0.348	*d* = 1.13
	Underwater speed (m/s)	1.72	(0.08)	*1.73*	*(0.09)*	0.878	*r* = 0.41	1.63	(0.07)	1.55	(0.05)	0.029	*d* = 1.97
Front crawl	Clean-swimming speed (m/s)	1.74	(0.03)	1.78	(0.02)	0.012	*d* = 2.41	1.55	(0.03)	1.57	(0.04)	0.276	*d* = 0.82
	Stroke rate (cycles/min)	45.77	(2.60)	45.84	(2.55)	0.992	*d* = 0.04	45.42	(1.42)	44.16	(2.49)	0.314	*d* = 0.88
	Distance per stroke (m/cycle)	2.30	(0.13)	2.35	(0.12)	0.677	*d* = 0.57	2.05	(0.09)	2.14	(0.13)	0.244	*d* = 1.14
	Underwater distance (m)	7.45	(0.85)	7.13	(1.32)	0.653	*d* = 0.40	*4.74*	*(1.57)*	5.36	(0.91)	0.535	*r* = 0.24
	Underwater time (s)	3.64	(0.47)	2.69	(0.64)	0.013	*d* = 2.40	*2.62*	*(0.88)*	2.21	(0.42)	0.115	*r* = 0.54
	Underwater speed (m/s)	2.05	(0.08)	2.69	(0.20)	<0.001	*d* = 5.99	1.77	(0.09)	2.43	(0.10)	<0.001	*d* = 9.31

**Table 2:** Results from a two-way analysis of variance on individual medley scores relative to the mean stroke-specialist scores. Information in bold font shows significant effects detected by the two-way ANOVA.

	Clean-Swimming Speed (%)	Stroke Rate (%)	Distance per Stroke (%)	Underwater Distance (%)	Underwater Time (%)	Underwater Speed (%)
Sex effect	*F* = 3.83	*F* = 1.78	*F* = 0.77	*F* = 0.004	*F* = 0.04	*F* = 0.27
	*η_p_*^2^ = 0.02	*η_p_*^2^ = 0.02	*η_p_*^2^ = 0.01	*η_p_*^2^ < 0.001	*η_p_*^2^ < 0.001	*η_p_*^2^ < 0.001
	*p* = 0.06	*p* = 0.06	*p* = 0.38	*p* = 0.95	*p* = 0.84	*p* = 0.60
Stroke effect	***F* = 25.04**	***F* = 7.14**	***F* = 16.05**	*F* = 1.58	***F* = 13.92**	***F* = 170.7**
	***η_p_*^2^ = 0.48**	***η_p_*^2^ = 0.27**	***η_p_*^2^ = 0.45**	*η_p_*^2^ =0.08	***η_p_*^2^ =0.42**	***η_p_*^2^ =0.88**
	***p* < 0.001**	***p* < 0.001**	***p* < 0.001**	*p* = 0.20	***p* < 0.001**	***p* < 0.001**
Interaction	***F* = 7.33**	*F* = 0.23	*F* = 0.73	*F* = 0.77	*F* = 0.53	***F* = 5.07**
	***η_p_*^2^ = 0.14**	*η_p_*^2^ = 0.01	*η_p_*^2^ = 0.02	*η_p_*^2^ =0.04	*η_p_*^2^ =0.02	***η_p_*^2^ =0.03**
	***p* < 0.001**	*p* = 0.87	*p* = 0.54	*p* = 0.52	*p* = 0.66	***p* = 0.004**

## Data Availability

The original contributions presented in the study are included in the article. Further inquiries can be directed to the corresponding author.
